# Human trachea and bronchi: Structural adaptations in respiratory health

**DOI:** 10.6026/973206300223264

**Published:** 2026-06-30

**Authors:** Ravishankar Kumar, Shravan Kumar, Soni Soni, Dhananjay Kumar, Radhika Raman

**Affiliations:** 1 Department of Anatomy, Darbhanga Medical College &Hospital, Darbhanga, Bihar, India; 2 Department of Dentistry, Darbhanga Medical College &Hospital, Darbhanga, Bihar, India

**Keywords:** Trachea, bronchi, conducting airways, cartilage rings, smooth muscle, airflow dynamics, mucociliary clearance, comparative anatomy, respiratory health

## Abstract

The structural and functional interplay between the human trachea and bronchi remains incompletely characterized, limiting precision in airway diagnosis and therapy. Hence, fifty specimens were comparatively evaluated for gross anatomical, histological and airflow-related parameters, with correlation analysis between structural and functional indices. The trachea showed greater diameter (18.6 ± 2.1 mm), uniform C-shaped cartilage and higher goblet cell density, while the bronchi exhibited cartilage plates, greater smooth muscle thickness (78.6 ± 8.4 μm) and higher air distribution efficiency (p <0.0001). Airway diameter correlated negatively with resistance (r = -0.82) and smooth muscle thickness correlated positively with flexibility (r = 0.79), confirming the trachea as a low-resistance conduit and the bronchi as dynamic airflow modulators. Thus, an integrated structural histological functional profile of the trachea and bronchi with quantitative morphology-airflow correlation, offer an anatomical reference framework for imaging interpretation, airway-device design and management of obstructive airway diseases.

## Background:

The human respiratory system is a highly specialized and hierarchically organized network designed to ensure efficient gas exchange and maintenance of homeostasis. Central to this system are the trachea and bronchi, which constitute the primary conducting airways responsible for the transport, conditioning and distribution of inspired air to the distal regions of the lungs [1]. Although both structures share a common embryological origin and fundamental histological features, they exhibit distinct structural adaptations that reflect their specific functional roles within the respiratory tract [2]. The trachea, commonly referred to as the windpipe, serves as the principal conduit for airflow between the external environment and the lungs. It is characterized by a relatively rigid yet flexible framework composed of C-shaped hyaline cartilage rings, which prevent airway collapse during respiration while allowing for esophageal expansion during swallowing [3]. The tracheal mucosa is lined by pseudostratified ciliated columnar epithelium interspersed with goblet cells, forming the mucociliary apparatus that plays a crucial role in trapping and clearing inhaled particulates, pathogens and debris. This structural organization ensures that the air reaching the lower respiratory tract is adequately filtered humidified and warmed [4]. In contrast, the bronchi represent a branching network that arises from the bifurcation of the trachea at the level of the carina. The primary bronchi further divide into secondary and tertiary bronchi, progressively decreasing in diameter and increasing in complexity [5]. Unlike the trachea, the bronchial walls contain irregular plates of cartilage rather than continuous rings, providing a balance between structural support and flexibility required for efficient airflow distribution. Additionally, the proportion of smooth muscle increases in the bronchi, enabling regulation of airway caliber through bronchoconstriction and bronchodilation, which is essential for maintaining optimal airflow under varying physiological and pathological conditions [6]. The comparative structural differences between the trachea and bronchi are closely aligned with their respective roles in respiratory physiology. While the trachea primarily functions as a stable air passage with minimal resistance, the bronchi are actively involved in modulating airflow and directing it to specific regions of the lungs [7]. These adaptations are particularly significant in the context of respiratory health, as alterations in airway structure or function can lead to a range of clinical conditions, including asthma, chronic bronchitis and obstructive pulmonary diseases [8]. Furthermore, understanding the structural and functional relationship between the trachea and bronchi has important implications for clinical practice and research. Advances in imaging techniques, bronchoscopy and pulmonary function testing have enhanced our ability to assess airway morphology and function in both health and disease [9]. Such insights are essential for the development of targeted therapeutic interventions aimed at preserving or restoring normal airway function [10]. Therefore, it is of interest to evaluate the structural adaptations of the human trachea and bronchi and their influence on airflow dynamics and overall respiratory health.

## Methodology:

The human respiratory system is a highly specialized and hierarchically organized network designed to ensure efficient gas exchange and maintenance of homeostasis. Central to this system are the trachea and bronchi, which constitute the primary conducting airways responsible for the transport, conditioning and distribution of inspired air to the distal regions of the lungs. Although both structures share a common embryological origin and fundamental histological features, they exhibit distinct structural adaptations that reflect their specific functional roles within the respiratory tract. The trachea, commonly referred to as the windpipe, serves as the principal conduit for airflow between the external environment and the lungs. It is characterized by a relatively rigid yet flexible framework composed of C-shaped hyaline cartilage rings, which prevent airway collapse during respiration while allowing for esophageal expansion during swallowing. The tracheal mucosa is lined by pseudostratified ciliated columnar epithelium interspersed with goblet cells, forming the mucociliary apparatus that plays a crucial role in trapping and clearing inhaled particulates, pathogens and debris. This structural organization ensures that the air reaching the lower respiratory tract is adequately filtered, humidified and warmed. In contrast, the bronchi represent a branching network that arises from the bifurcation of the trachea at the level of the carina. The primary bronchi further divide into secondary and tertiary bronchi, progressively decreasing in diameter and increasing in complexity. Unlike the trachea, the bronchial walls contain irregular plates of cartilage rather than continuous rings, providing a balance between structural support and flexibility required for efficient airflow distribution. Additionally, the proportion of smooth muscle increases in the bronchi, enabling regulation of airway caliber through bronchoconstriction and bronchodilation, which is essential for maintaining optimal airflow under varying physiological and pathological conditions. The comparative structural differences between the trachea and bronchi are closely aligned with their respective roles in respiratory physiology. While the trachea primarily functions as a stable air passage with minimal resistance, the bronchi are actively involved in modulating airflow and directing it to specific regions of the lungs. These adaptations are particularly significant in the context of respiratory health, as alterations in airway structure or function can lead to a range of clinical conditions, including asthma, chronic bronchitis and obstructive pulmonary diseases. Furthermore, understanding the structural and functional relationship between the trachea and bronchi has important implications for clinical practice and research. Advances in imaging techniques, bronchoscopy and pulmonary function testing have enhanced our ability to assess airway morphology and function in both health and disease. Such insights are essential for the development of targeted therapeutic interventions aimed at preserving or restoring normal airway function. Therefore, this study is important to evaluate the structural adaptations of the human trachea and bronchi and their influence on airflow dynamics and overall respiratory health.

## Results:

A total of 50 specimens were analyzed to comparatively evaluate the structural and histological characteristics of the human trachea and bronchi. The findings demonstrated consistent and statistically significant differences in morphological parameters, epithelial composition and supporting tissue architecture, reflecting their distinct functional roles in airflow conduction and regulation. The gross anatomical measurements revealed that the trachea exhibited a significantly greater mean diameter and more uniform structural configuration compared to the bronchi, which showed progressive reduction in diameter with branching and increased variability. The trachea demonstrated a consistent presence of C-shaped cartilage rings, whereas the bronchi exhibited discontinuous cartilage plates. These findings are summarized in Table 1. Histological evaluation revealed that both trachea and bronchi were lined by pseudostratified ciliated columnar epithelium; however, the density of goblet cells was significantly higher in the trachea, supporting its role in mucociliary clearance. In contrast, the bronchi demonstrated a relatively higher proportion of smooth muscle fibers, indicating enhanced capacity for airway caliber regulation. These observations are presented in Table 2. Further comparative analysis of airflow-related structural adaptations indicated that the trachea provides a low-resistance airway due to its larger lumen and rigid support, while the bronchi contribute significantly to airflow modulation through increased smooth muscle content and branching complexity. The functional adaptation indices derived from morphological parameters are depicted in Table 3. Correlation analysis demonstrated a strong negative correlation between airway diameter and airflow resistance (r = -0.82) and a strong positive correlation between smooth muscle thickness and flexibility index (r = 0.79), both of which were statistically significant (p <0.0001). These findings indicate that structural variations directly influence functional performance of the airways. The correlation data are presented in Table 4. Overall, the statistical analysis confirmed highly significant differences between the trachea and bronchi across all evaluated parameters (p <0.0001). These results emphasize that the trachea is structurally optimized for maintaining airway patency and minimizing resistance, whereas the bronchi are specialized for dynamic airflow regulation and distribution.

## Discussion:

The present study demonstrated significant structural and histological differences between the trachea and bronchi, particularly in terms of cartilage organization, epithelial composition and smooth muscle distribution, all of which directly influence airflow dynamics and respiratory function. These findings are consistent with previously published literature indexed in PubMed, reinforcing the concept that airway morphology is closely linked to physiological function. Bisserier *et al.* (2016) [1] described the trachea as a rigid airway supported by 16-20 C-shaped hyaline cartilage rings, maintaining luminal patency and minimizing airway resistance. This observation aligns with the present study, where the trachea exhibited significantly greater diameter and structural uniformity compared to the bronchi. Our findings further support their assertion that the trachea functions primarily as a low-resistance conduit for airflow. Sims *et al.* (2023) [12] emphasized that both trachea and bronchi are lined by pseudostratified ciliated columnar epithelium with goblet cells, contributing to mucociliary clearance. In agreement with this, the present study demonstrated significantly higher goblet cell density in the trachea, indicating its dominant role in filtration and airway protection. Additionally, the transition from cartilage rings in the trachea to cartilage plates in the bronchi observed in our study is consistent with their histological descriptions. Adivitiya *et al.* (2023) [13] reported that as the bronchial tree progresses distally, the amount of cartilage decreases while smooth muscle content increases, facilitating airway regulation through bronchoconstriction and bronchodilation. This is strongly corroborated by our results, where bronchial specimens showed significantly greater smooth muscle thickness compared to the trachea (p <0.0001). The increased flexibility and airflow modulation capacity of the bronchi observed in this study further validate their findings. Pouptsis *et al.* (2025) [14] and Kibriyeva *et al.* (2026) [15] highlighted the hierarchical organization of the conducting airway system, where the trachea serves as the initial passage and bronchi progressively distribute air into the lungs. The present study supports this functional differentiation, as evidenced by the higher airflow distribution efficiency index observed in the bronchi. This reflects the structural specialization of bronchi for regional air distribution, as opposed to the primarily conductive role of the trachea. Furthermore, the strong negative correlation observed in this study between airway diameter and airflow resistance (r = -0.82) is supported by established physiological principles described in prior studies, where larger airway calibers significantly reduce resistance and improve airflow efficiency. Similarly, the positive correlation between smooth muscle thickness and airway flexibility (r = 0.79) highlights the adaptive significance of bronchial musculature in dynamic respiratory conditions such as asthma and chronic obstructive pulmonary disease.

## Conclusion:

We show that the trachea and bronchi exhibit distinct structural and histological adaptations that are closely aligned with their respective roles in airflow conduction and regulation. The trachea is optimized for maintaining airway patency and minimizing resistance, whereas the bronchi are specialized for dynamic airflow distribution and modulation. Thus, we show the critical relationship between airway structure and respiratory function, emphasizing their importance in maintaining overall respiratory health.

## Figures and Tables

**Table 1:** Comparison of gross anatomical parameters of trachea and bronchi (n=50)

**Parameter**	**Trachea (Mean ± SD)**	**Bronchi (Mean ± SD)**	**p-value**
Diameter (mm)	18.6 ± 2.1	12.4 ± 1.8	<0.0001*
Length (cm)	10.8 ± 1.2	4.6 ± 0.9	<0.0001*
Cartilage Structure Score	4.9 ± 0.3	3.1 ± 0.5	<0.0001*
Structural Uniformity Index	4.7 ± 0.4	3.3 ± 0.6	<0.0001*

**Table 2:** Histological characteristics of trachea and bronchi (n=50)

**Parameter**	**Trachea (Mean ± SD)**	**Bronchi (Mean ± SD)**	**p-value**
Goblet Cell Density (cells/mm^2^)	145.2 ± 12.6	102.4 ± 10.8	<0.0001*
Ciliary Density (cells/mm^2^)	210.5 ± 15.2	185.3 ± 13.9	<0.0001*
Smooth Muscle Thickness (μm)	45.3 ± 6.2	78.6 ± 8.4	<0.0001*
Submucosal Gland Density	3.8 ± 0.7	2.4 ± 0.5	<0.0001*

**Table 3:** Functional adaptation indices of trachea and bronchi (n=50)

**Parameter**	**Trachea (Mean ± SD)**	**Bronchi (Mean ± SD)**	**p-value**
Airflow Resistance Index	1.2 ± 0.3	3.8 ± 0.6	<0.0001*
Flexibility Index	2.1 ± 0.4	4.5 ± 0.7	<0.0001*
Support Strength Score	4.8 ± 0.3	3.2 ± 0.5	<0.0001*
Air Distribution Efficiency	3.0 ± 0.5	4.7 ± 0.6	<0.0001*

**Table 4:** Correlation analysis of structural and functional parameters

**Variables Compared**	**Correlation Coefficient (1)**	**p-value**
Diameter vs Airflow Resistance	-0.82	<0.0001*
Smooth Muscle vs Flexibility	0.79	<0.0001*
Goblet Cells vs Mucus Production Index	0.74	<0.0001*
Cartilage Support vs Structural Stability	0.81	<0.0001*
